# Intensive Rural Medical Inspection

**Published:** 1913-09

**Authors:** 


### Intensive Rural Medical Inspection.

The feasibility of, as well as the necessity for, complete medical inspection of rural schools has been effectually demonstrated in a survey of all the schools in one county recently conducted by the State Board of Health of Virginia. An average county was selected, and in less than two months time every child in attendance in fifty schools was carefully examined. The negro schools and school children were as thoroughly inspected as the white.

The physical characteristics of the school buildings, and surroundings were first noted, such as air space, light space, heating and ventilating facilities, equipment as to sanitation and water supply, then the children were lined up, their height and weight were first taken, then their eyes, ears, nose, throat glands, skin, heart and lungs were carefully examined. All previous diseases from which they had suffered were also noted. A specially interesting point was the preliminary step then taken at every school looking toward the detailed examination of every child for intestinal parasites.

A tabulation of all the facts gathered in this survey is now being made, and a report will be submitted to the International Congress on School Hygiene which meets in Buffalo, August 25-30. Dr. Roy K. Flannagan, Director of Inspections for the Virginia State Board of Health, who conducted this investigation, will discuss the methods employed, and Dr. Allen W. Freeman, Assistant Commissioner of Health of Virginia and Director of the Rockefeller Hookworm Campaign in the State, will discuss other phases and facts developed.

Such a complete medical survey as this has never hitherto so far as known, been attempted in this country, and the results should prove exceptionally interesting.

Correcting unhygienic conditions and properly educating the school children in the first step in positive eugenics. Environment develops the qualities possessed by the child through his inheritance. We can not rightly judge who are the unfit until education has first tried to remove the dross.

